# Mapping the Structural Core of Human Cerebral Cortex

**DOI:** 10.1371/journal.pbio.0060159

**Published:** 2008-07-01

**Authors:** Patric Hagmann, Leila Cammoun, Xavier Gigandet, Reto Meuli, Christopher J Honey, Van J Wedeen, Olaf Sporns

**Affiliations:** 1 Department of Radiology, University Hospital Center and University of Lausanne (CHUV), Lausanne, Switzerland; 2 Signal Processing Laboratory (LTS5), Ecole Polytechnique Fédérale de Lausanne (EPFL), Lausanne, Switzerland; 3 Department of Psychological and Brain Sciences, Indiana University, Bloomington, Indiana, United States of America; 4 Martinos Center for Biomedical Imaging, Department of Radiology, Massachusetts General Hospital and Harvard Medical School, Boston, Massachusetts, United States of America; University College London, United Kingdom

## Abstract

Structurally segregated and functionally specialized regions of the human cerebral cortex are interconnected by a dense network of cortico-cortical axonal pathways. By using diffusion spectrum imaging, we noninvasively mapped these pathways within and across cortical hemispheres in individual human participants. An analysis of the resulting large-scale structural brain networks reveals a structural core within posterior medial and parietal cerebral cortex, as well as several distinct temporal and frontal modules. Brain regions within the structural core share high degree, strength, and betweenness centrality, and they constitute connector hubs that link all major structural modules. The structural core contains brain regions that form the posterior components of the human default network. Looking both within and outside of core regions, we observed a substantial correspondence between structural connectivity and resting-state functional connectivity measured in the same participants. The spatial and topological centrality of the core within cortex suggests an important role in functional integration.

## Author Summary

In the human brain, neural activation patterns are shaped by the underlying structural connections that form a dense network of fiber pathways linking all regions of the cerebral cortex. Using diffusion imaging techniques, which allow the noninvasive mapping of fiber pathways, we constructed connection maps covering the entire cortical surface. Computational analyses of the resulting complex brain network reveal regions of cortex that are highly connected and highly central, forming a structural core of the human brain. Key components of the core are portions of posterior medial cortex that are known to be highly activated at rest, when the brain is not engaged in a cognitively demanding task. Because we were interested in how brain structure relates to brain function, we also recorded brain activation patterns from the same participant group. We found that structural connection patterns and functional interactions between regions of cortex were significantly correlated. Based on our findings, we suggest that the structural core of the brain may have a central role in integrating information across functionally segregated brain regions.

## Abstract 3

Mapping of major structural connections of the human cortex reveals a core of brain regions that are highly interconnected and highly central with respect to the rest of the brain.

## Introduction

Human cerebral cortex consists of approximately 10^10^ neurons that are organized into a complex network of local circuits and long-range fiber pathways. This complex network forms the structural substrate for distributed interactions among specialized brain systems [1–3]. Computational network analysis [4] has provided insight into the organization of large-scale cortical connectivity in several species, including rat, cat, and macaque monkey [4–7]. In human cortex, the topology of functional connectivity patterns has recently been investigated [8–11], and key attributes of these patterns have been characterized across different conditions of rest or cognitive load. A major feature of cortical functional connectivity is the default network [12–18], a set of dynamically coupled brain regions that are found to be more highly activated at rest than during the performance of cognitively demanding tasks. Spontaneous functional connectivity resembling that of the human default network was reported in the anaesthetized macaque monkey, and functional connectivity patterns in the oculomotor system were found to correspond to known structural connectivity [19]. Computational modeling of spontaneous neural activity in large-scale cortical networks of the macaque monkey has indicated that anti-correlated activity of regional clusters may reflect structural modules present within the network [20]. These studies suggest that, within cerebral cortex, structural modules shape large-scale functional connectivity.

Understanding the structural basis of functional connectivity patterns requires a comprehensive map of structural connection patterns of the human brain (the human connectome [1]). Recent advances in diffusion imaging and tractography methods permit the noninvasive mapping of white matter cortico-cortical projections at high spatial resolution [21–25], yielding a connection matrix of inter-regional cortical connectivity [26–29]. Previous studies have demonstrated small-world attributes and exponential degree distributions within such structural human brain networks [26,27]. In the present study, using diffusion spectrum imaging (DSI) we derived high-resolution cortical connection matrices and applied network analysis techniques to identify structural modules. Several techniques reveal the existence of a set of posterior medial and parietal cortical regions that form a densely interconnected and topologically central core. The structural core contains numerous connector hubs, and these areas link the core with modules in temporal and frontal cortex. A comparison of diffusion imaging and resting state functional MRI (fMRI) data reveals a close relationship between structural and functional connections, including for regions that form the structural core. We finally discuss anatomical and functional imaging data, suggesting an important role for the core in cerebral information integration.

## Results

### Datasets and Network Measures

**Figure 1:**
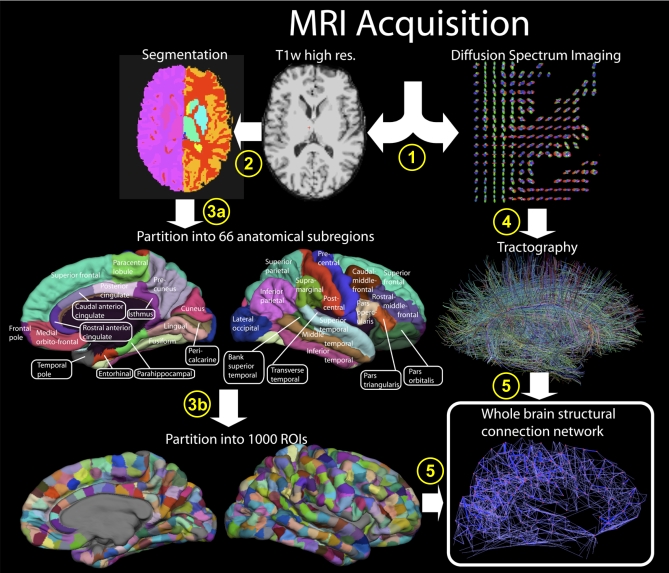
Extraction of a Whole Brain Structural Connectivity Network (1) High-resolution T1 weighted and diffusion spectrum MRI (DSI) is acquired. DSI is represented with a zoom on the axial slice of the reconstructed diffusion map, showing an orientation distribution function at each position represented by a deformed sphere whose radius codes for diffusion intensity. Blue codes for the head-feet, red for left-right, and green for anterior-posterior orientations. (2) White and gray matter segmentation is performed from the T1-weighted image. (3a) 66 cortical regions with clear anatomical landmarks are created and then (3b) individually subdivided into small regions of interest (ROIs) resulting in 998 ROIs. (4) Whole brain tractography is performed providing an estimate of axonal trajectories across the entire white matter. (5) ROIs identified in step (3b) are combined with result of step (4) in order to compute the connection weight between each pair of ROIs. The result is a weighted network of structural connectivity across the entire brain. In the paper, the 66 cortical regions are labeled as follows: each label consists of two parts, a prefix for the cortical hemisphere (r = right hemisphere, l = left hemisphere) and one of 33 designators: BSTS = bank of the superior temporal sulcus, CAC = caudal anterior cingulate cortex, CMF = caudal middle frontal cortex, CUN = cuneus, ENT = entorhinal cortex, FP = frontal pole, FUS = fusiform gyrus, IP = inferior parietal cortex, IT = inferior temporal cortex, ISTC = isthmus of the cingulate cortex, LOCC = lateral occipital cortex, LOF = lateral orbitofrontal cortex, LING = lingual gyrus, MOF = medial orbitofrontal cortex, MT = middle temporal cortex, PARC = paracentral lobule, PARH = parahippocampal cortex, POPE = pars opercularis, PORB = pars orbitalis, PTRI = pars triangularis, PCAL = pericalcarine cortex, PSTS = postcentral gyrus, PC = posterior cingulate cortex, PREC = precentral gyrus, PCUN = precuneus, RAC = rostral anterior cingulate cortex, RMF = rostral middle frontal cortex, SF = superior frontal cortex, SP = superior parietal cortex, ST = superior temporal cortex, SMAR = supramarginal gyrus, TP = temporal pole, and TT = transverse temporal cortex.

Network analyses were carried out for high-resolution connection matrices (*n* = 998 regions of interest [ROIs] with an average size of 1.5 cm^2^), as well as for regional connection matrices (*n* = 66 anatomical subregions) (see Methods and Figure 1). All networks covered the entire cortices of both hemispheres but excluded subcortical nodes and connections. When not indicated otherwise, the data shown in this paper are based on the analysis of individual high-resolution connection matrices, followed by averaging across five human participants.

Network measures included degree, strength, betweenness centrality, and efficiency (see Methods). Briefly, degree and strength of a given node measure the extent to which the node is connected to the rest of the network, while centrality and efficiency capture how many short paths between other parts of the network pass through the node. A node with high degree makes many connections (where each connection is counted once), while a node with high strength makes strong connections (where strength is equal to the sum of connection density or weight). A node with high betweenness centrality lies on many of the shortest paths that link other nodes in the network to one another. A node with high efficiency is itself found to be, on average, at a short distance from other nodes in the network.

### Degree and Strength Distribution

**Figure 2:**
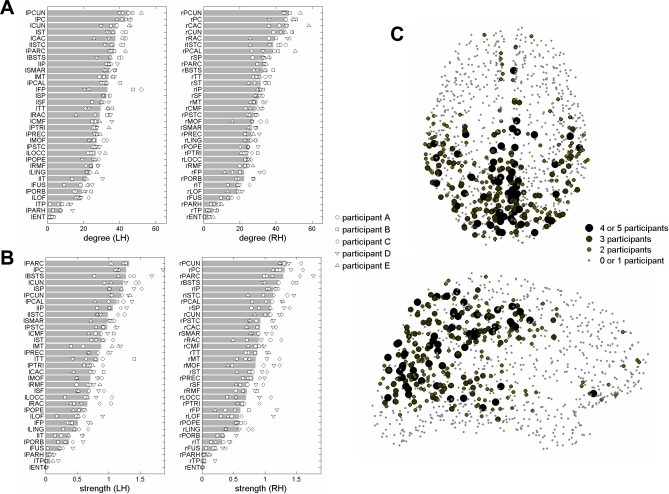
Node Degree and Node Strength Distributions (A) Ranked distribution of node degree for left and right cerebral hemispheres. Shaded bars represent means across five participants and symbols indicate data for individual participants. (B) Ranked distribution of node strength for left and right cerebral hemispheres. (C) ROI strength obtained from high-resolution connection matrices. The plot shows how consistently ROI strength ranked in the top 20% across participants.

We found binary, high-resolution brain networks to be sparsely connected, with connection densities varying between 2.8% and 3.0%. Between 9% and 14% of all binary connections were interhemispheric. 54% of the total edge mass (the sum of all fiber densities) was accounted for by connections linking ROIs belonging to the same anatomical subregion, 42% was made between ROIs belonging to different anatomical subregions located in the same cortical hemisphere, and 4% was interhemispheric (homotopic or heterotopic). Confirming earlier reports [25], we found that cumulative distributions of node degree and node strength (Figure S1) were exponential rather than scale-free. While not scale-free, node degrees and node strengths for single ROIs can vary over a significant range (approximately 10-fold), indicating that fiber densities are not uniformly distributed across the cortical surface. Figure 2A and 2B shows the distribution of average node degree and node strength rank-ordered by anatomical subregion. A large number of ROIs with high degree and high strength are localized within subregions of medial cortex (e.g., cuneus and precuneus, posterior and anterior cingulate cortex) and temporal cortex (e.g., bank of the superior temporal sulcus). A plot of the distribution of node strengths on the cortical surface across all participants (Figure 2C) shows consistently high values in posterior medial cortex, in medial frontal cortex, and in superior temporal cortex. In addition, we found evidence for positive assortativity (Text S1) and small-world attributes (Text S2).

### Network Visualizations

**Figure 3:**
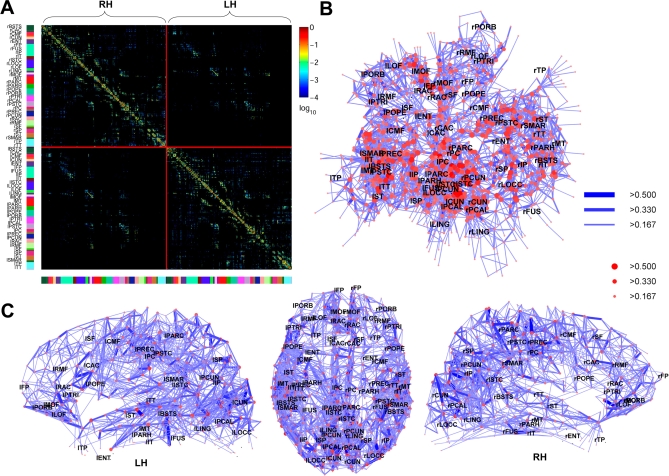
High-Resolution Connection Matrix, Network Layout and Connectivity Backbone (Participant A, scan 2) (A) Matrix of fiber densities (connection weights) between all pairs of *n* = 998 ROIs. ROIs are plotted by cerebral hemispheres, with right-hemispheric ROIs in the upper left quadrant, left-hemispheric ROIs in the lower right quadrant, and interhemispheric connections in the upper right and lower left quadrants. The color bars at the left and bottom of the matrix correspond to the colors of the 66 anatomical subregions shown in Figure 1. All connections are symmetric and displayed with a logarithmic color map. (B) Kamada-Kawai force-spring layout of the connectivity backbone. Labels indicating anatomical subregions are placed at their respective centers of mass. Nodes (individual ROIs) are coded according to strength and edges are coded according to connection weight (see legend). (C) Dorsal and lateral views of the connectivity backbone. Node and edge coding as in (B).

A representative example of a high-resolution structural connection matrix of an individual human brain is shown in Figure 3A. Entries of the matrix represent fiber densities between pairs of single ROIs. The matrix shown in the example displays a total of 14,865 symmetric connections (connection density 3.0%). To visualize structural patterns within this connection matrix, we extracted the connectivity backbone ([30], see Methods), which is displayed in Figure 3B with a layout derived from the Kamada-Kawai force-spring algorithm [31] implemented in Pajek [32]. The algorithm generates a spatial arrangement of ROIs along clearly defined anterior-posterior and medial-lateral axes and reveals clusters of dense connectivity within posterior, temporal, and frontal cortex. Figure 3C shows the connectivity backbone plotted in anatomical coordinates. The dorsal view shows groupings of highly interconnected clusters of ROIs arranged along the medial cortical surface, extending from the precuneus via posterior and anterior cingulate cortex to the medial orbitofrontal cortex. Dorsal and lateral views additionally show clusters of temporal and frontal ROIs in both hemispheres.

**Figure 4:**
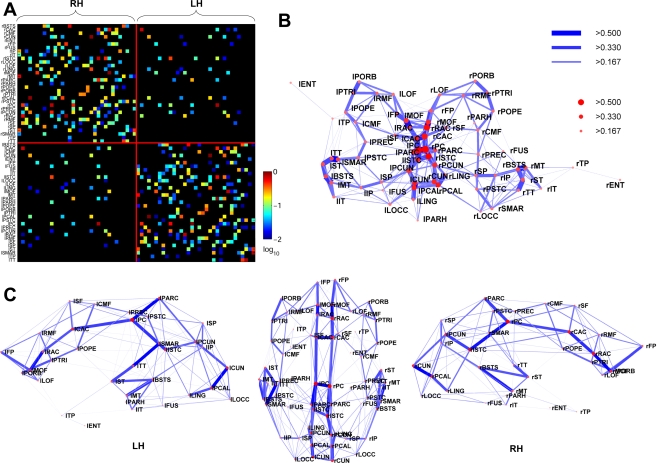
Average Regional Connection Matrix, Network Layout, and Connectivity Backbone (A) Matrix of inter-regional fiber densities between pairs of anatomical subregions, obtained by averaging over fiber densities for all pairs of ROIs within the regions, and averaging across all five participants. Connection weights are symmetric and are plotted on a logarithmic scale. For corresponding plots for all individual participants, see Figure S2. (B) Network layout. (C) Dorsal and medial views of the connectivity backbone in anatomical coordinates.

Major structural patterns become more evident when considering the average regional connection matrix (Figure 4A). The matrix is constructed by calculating mean fiber densities over individual pairs of ROIs comprising each subregion, followed by the averaging of densities over all five participants. Regional connection matrices for each individual participant are shown in Figure S2. Figure 4B displays the connectivity backbone constructed from the average regional connection matrix, revealing groupings of anatomical regions largely corresponding to those shown for the high-resolution backbone in Figure 3B. A dominant feature of the regional connection matrix is a single, callosally interconnected cluster of regions extending from the cuneus and precuneus via cingulate cortex to medial frontal cortex. In addition, each hemisphere contains a single, relatively distinct cluster of temporal cortical regions, as well as a less-densely interconnected frontal cluster comprising periorbital cortex, pars opercularis, pars triangularis, and other regions.

### k-Core Decomposition, Modularity, and Hubs

While network visualization provides strong hints of connectional relationships, objective methods are needed to map structural cores, to delineate network modules, and to identify hub regions that link distinct clusters. We quantified these phenomena using k-core decomposition [33], spectral community detection [34], and nodal participation indices [35], respectively.

**Figure 5:**
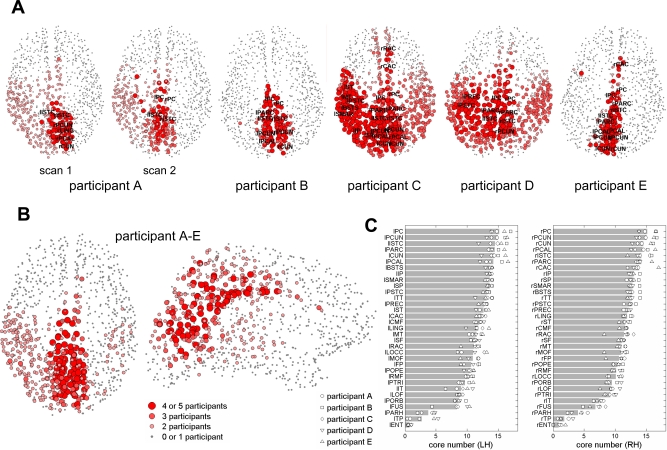
Structural Network Cores (A) Network cores for each individual participant derived by k-core decomposition of a binary connection matrix obtained by thresholding the high-resolution fiber densities such that a total of 10,000 connections remain in each participant. Nodes are plotted according to their core number, counted backwards from the last remaining core. (B) Average network core across all five participants. (C) Ranked distribution of core numbers for left and right cerebral hemispheres. Shaded bars represent means across five participants and symbols indicate data for individual participants

Intuitively, a network core is a set of nodes that are highly and mutually interconnected. For a binary network, the k-core is the largest subgraph comprising nodes of degree at least k, and is derived by recursively peeling off nodes with degree lower than k until none remain [33]. Each node is then assigned a core number, which is defined as the largest k such that the node is still contained in the k-core. We performed k-core decomposition on binary, high-resolution connection matrices from all five participants and derived the core number for each ROI, as well as the average core number for each anatomical subregion (Figure 5). A large core number indicates that an ROI or region is resistant to this erosive procedure and participates in high-k structural cores of the network. In all participants, full erosion occurs at a core number of ∼20. The most consistent members of the highest degree k-core for each network (Figure 5A and 5B) were the precuneus, the posterior cingulate, the isthmus of the cingulate, and the paracentral lobule in both hemispheres. In all participants, the structural core was located within posterior medial cortex, and often extended laterally into parietal and temporal cortices, especially in the left hemisphere. A rank-ordered distribution of average core numbers per anatomical subregion (Figure 5C) identifies the posterior cingulate cortex, the isthmus of the cingulate cortex, the precuneus, the cuneus, and the paracentral lobule as regions with a high core number. Several temporal and parietal structures, including the superior and inferior parietal cortex, the bank of the superior temporal gyrus, and transverse temporal cortex all have high core rankings as well. k-Core decomposition, as applied in our study, largely discards edge weights. To test if the inclusion of edge weight information would alter our conclusions, we designed a procedure that operates on the weighted fiber density matrix and erodes vertices according to their strength (“s-core decomposition”). s-Core decomposition (Figure S3) identified the posterior cingulate cortex, the precuneus, the cuneus, the paracentral lobule, as well as the superior and inferior parietal cortex, all in both hemispheres, as members of the structural core.

**Figure 6:**
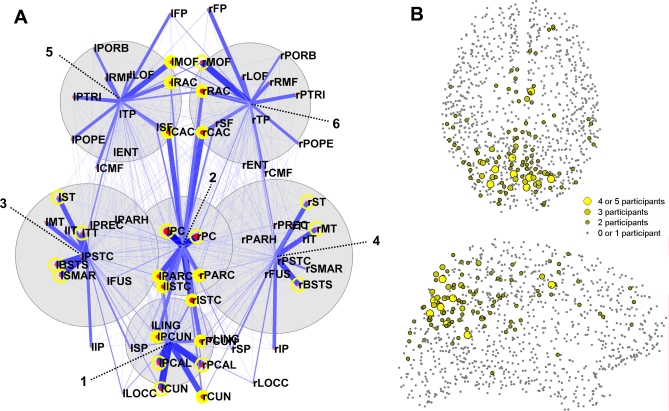
Modularity and Hub Classification The modularity was derived from the average regional connection matrix. Modules are listed in Table S1. (A) The plot shows a dorsal view, with nodes representing anatomical subregions. The spatial position of each region corresponds to the center of mass coordinates calculated from participant A, scan 2 (as seen in Figure 4C). Six modules are shown as gray circles centered on their center of mass and sized according to their number of members. Edges correspond to the average connection densities of each region with the member regions of each of the six modules, plotted between that region's spatial coordinates and the center of mass of each module Connector hubs are defined as regions with above average strength and a participation index *p* ≥ 0.3, indicating a high proportion of cross-module connectivity. These regions are marked as filled yellow circles. Provincial hubs have above-average strength and *P* < 0.3; they are marked as unfilled yellow circles. (B) Connector hubs obtained from analyses of high-resolution connection matrices. ROIs are displayed according to how consistently a given ROI was identified as a connector hub across participants.

We used spectral graph partitioning [34] to identify modules within the weighted high-resolution (*n* = 998) network as well as within the weighted average regional (*n* = 66) network. The spectral algorithm provides a means of grouping regions in a way that optimally matches the intrinsic modularity of the network. Optimal modularity for the average regional connectivity matrix was achieved with six clusters (Figure 6A and Table S1). Four contralaterally matched modules were localized to frontal and temporo-parietal areas of a single hemisphere. The two remaining modules comprised regions of bilateral medial cortex, one centered on the posterior cingulate cortex and another centered on the precuneus and pericalcarine cortex. Recovering the modularity structure using high-resolution connection matrices produced similar results (unpublished data).

Knowledge of the distribution of connections within and between modules enabled us to identify provincial hubs (hub regions that are highly connected within one module) and connector hubs (hub regions that link multiple modules) [35]. Without exception, connector hubs are located within the anterior-posterior medial axis of the cortex (Figure 6A), including bilaterally the rostral and caudal anterior cingulate, the paracentral lobule, and the precuneus. Examination of high-resolution connection matrices shows that the majority of connector hub ROIs is consistently found in posterior medial and parietal cortex (Figure 6B). Provincial hubs are members of the frontal (e.g., medioorbitofrontal cortex), temporoparietal (e.g., bank of the superior temporal sulcus, superior temporal cortex) or occipital modules (e.g., pericalcarine cortex). Most core regions, as identified by k-core or s-core decomposition, are members of the two medial modules. When combined into a single “core module,” over 70% of the between-module edge mass is attached to the core.

When modularity detection was applied to more restricted portions of the high-resolution connection datasets, for example the visual and frontal cortex, we were able to recover clusters that were consistent with those found in previous studies based on classical anatomical techniques, or orderings that were suggested based on functional subdivisions. For example, we found, in all five participants, a segregated dorsal and ventral cluster of visual ROIs, corresponding in location and extent to the dorsal and ventral stream of visual cortex [36]. Clustering of frontal cortical ROIs yielded distinct clusters centered on orbital, medial, and lateral frontal cortex (Figure S4).

### Centrality and Efficiency

**Figure 7:**
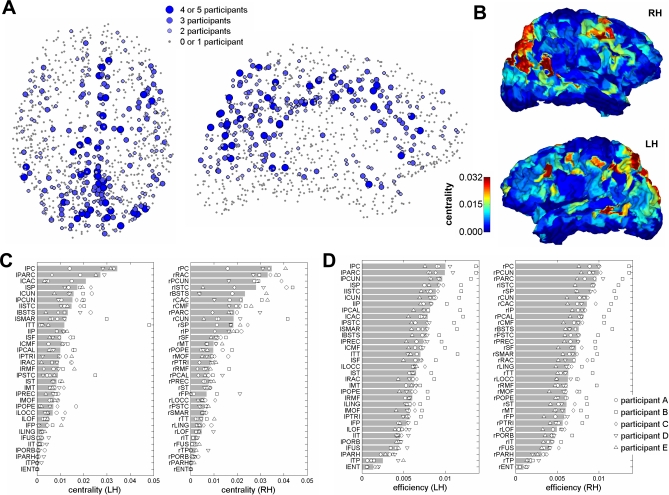
Centrality and Efficiency (A) ROI centrality obtained from analyses of high-resolution connection matrices. The plot shows how consistently ROI centrality ranked in the top 20% across participants. (B) Lateral views of the right and left cerebral hemispheres showing ROI centrality, averaged across all five participants and projected onto the cortical surface of participant A. (C) Ranked distribution of betweenness centrality for left and right cerebral hemispheres. Shaded bars represent means across five participants and symbols indicate data for individual participants. (D) Ranked distribution of efficiency for left and right cerebral hemispheres.

Regions with elevated betweenness centrality are positioned on a high proportion of short paths within the network [37]. The spatial distribution of ROIs with high betweenness centrality (Figure 7A and 7B) shows high centrality for regions of medial cortex such as the precuneus and posterior cingulate cortex, as well as for portions of medial orbitofrontal cortex, inferior and superior parietal cortex, as well as portions of frontal cortex. Figure 7B provides lateral views of the distribution of centrality across the two cerebral hemispheres showing that ROIs with high centrality are widely distributed. For example, ROIs with high centrality are found in the superior and middle frontal gyrus, in the inferior and superior parietal cortex, in addition to in regions of cingulate and medial posterior cortex (Table S2). Averaged over all ROIs belonging to the same anatomical subdivision and over all participants (Figure 7C), centrality appears highest in the right and left posterior cingulate cortex, as well as other subdivisions of cingulate cortex, and the precuneus and cuneus. Efficiency is related to closeness centrality, in that regions with high efficiency maintain short average path lengths with other regions in the network. We find that the posterior cingulate cortex, the precuneus, and the paracentral lobule are most highly ranked in both cerebral hemispheres (Figure 7D).

### Validation of Structural Imaging

Five lines of evidence support the robustness and validity of the diffusion imaging and tractography methodology applied in this paper (see also Text S3). First, within-participant interhemispheric differences in structural connections were modest, since the connection patterns between left and right cortical hemispheres were highly correlated (*r* ^2^ = 0.94, *p* < 10^−10^, Figure S2). This indicates methodological consistency within individual scanning sessions. Second, two scans of participant A performed several days apart yielded highly consistent regional connection matrices (*r* ^2^ = 0.78, *p* < 10^−10^, Figure S2). Third, we found that after introducing random perturbations of the structural connection matrix that fractionally degraded the connection pattern, our network measures were consistent with those reported for the intact connectivity, indicating that our main conclusions were insensitive to low levels of homogeneous noise potentially introduced in either scanning or tractography (Figure S6).

Fourth, we collected diffusion imaging data from a single hemisphere of macaque cortex to compare connection data obtained by diffusion spectrum imaging to connection data obtained by anatomical tract tracing (see Text S4). An overlay of structural connectivity derived by DSI and a macaque anatomical connection matrix derived from Cocomac data [20] is shown in Figure S9. We found that 78.9% of all DSI fibers were identified in positions where connections had been identified by tract tracing methods and recorded in Cocomac. A further 15.0% were placed in positions where the presence or absence of a pathway is currently unknown. The remaining 6.1% were placed in positions where connections had been reported to be absent.

**Figure 8:**
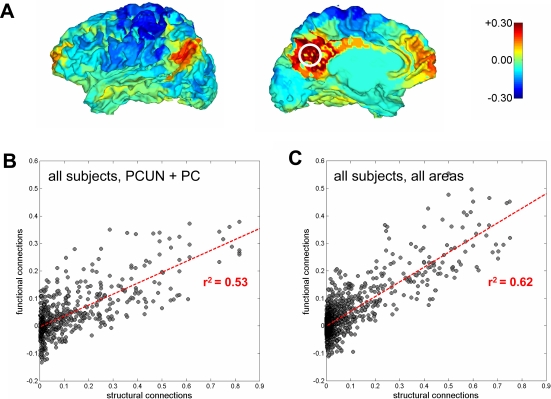
Comparison of Structural and Functional Connectivity (A) Map of functional correlations from resting state fMRI for a cluster of five seed ROIs located within 10 mm of the Talairach coordinate [–5 −49 40] (marked by a white circle). Correlations are averaged over the five ROIs and over scanning sessions for all five participants. The plot shows a lateral and medial view of the left cerebral hemisphere. (B) Scatter plot of structural and functional connections of the precuneus and posterior cingulate cortex (PCUN and PC, left and right hemisphere), for all five participants. (C) Scatter plots for structural and functional connections averaged over all five participants, for all anatomical subregions in both hemispheres.

Fifth, we performed resting state fMRI in all five participants to derive networks of functional connections and to investigate the degree to which structural connections and functional connections are correlated. Figure 8A shows a map of the functional connections averaged over all five participants plotted for a group of five seed ROIs, all of which were within 10 mm of the Talairach coordinate [–5 −49 40], which is located within the precuneus and posterior cingulate and was used in a previous study [17] to map the brain's default network (see also the seed region ‘PCC' in Figure 1 of [17]). Consistent with earlier observations (e.g., [15,17,18]), we find that this seed region maintains positive functional connections with portions of posterior medial cortex, medial orbitofrontal cortex, and lateral parietal cortex. Figure 8B shows a scatter plot of structural connections and functional connections for the precuneus and the posterior cingulate cortex (both hemispheres, all participants). The plot indicates that the strengths of structural connections as estimated from diffusion imaging are highly predictive of the strengths of functional connections (*r* ^2^ = 0.53, *p* < 10^−10^). Scatter plots of structural connections and functional connections for all anatomical subregions averaged over all five participants (Figure 8C) also reveal significant correlations between their strengths (*r* ^2^ = 0.62, *p* < 10^−10^). Figure 8B and 8C demonstrate that stronger DSI connections are quantitatively predictive of stronger functional connectivity. The results from this comparison of structural and functional connections support the validity of the DSI-derived structural connection patterns and suggest that structural connections identified by DSI do, in fact, participate in shaping the functional topology of the default network.

## Discussion

Cortical connectivity plays a crucial role in shaping spontaneous and evoked neural dynamics. We mapped structural cortico-cortical pathways in the human cerebral cortex at high spatial resolution and found evidence for the existence of a structural core composed of posterior medial and parietal cortical regions that are densely interconnected and topologically central.

We characterize the structural core by mapping network indices, such as node degree, strength, and centrality, and by applying several network analysis methods: extracting a structural backbone, performing core decomposition, retrieving network modules, and classifying hub nodes . While several of these measures are known to be interrelated, each provides a different viewpoint from which to discern major features of the large-scale architecture. Based on their aggregated ranking scores across six network measures (Table 1), we identified eight anatomical subregions as members of the structural core. These are the posterior cingulate cortex, the precuneus, the cuneus, the paracentral lobule, the isthmus of the cingulate, the banks of the superior temporal sulcus, and the inferior and superior parietal cortex, all of them in both hemispheres. These regions are chosen because they exhibit elevated fiber counts and densities (node degree and strength), they are most resistant to the erosive procedures of k-core and s-core decomposition and they have high topological centrality. The high degree of interhemispheric coupling within the core further suggests that it acts as a single integrated system from which processes in both cortical hemispheres are coordinated.

**Table 1:**
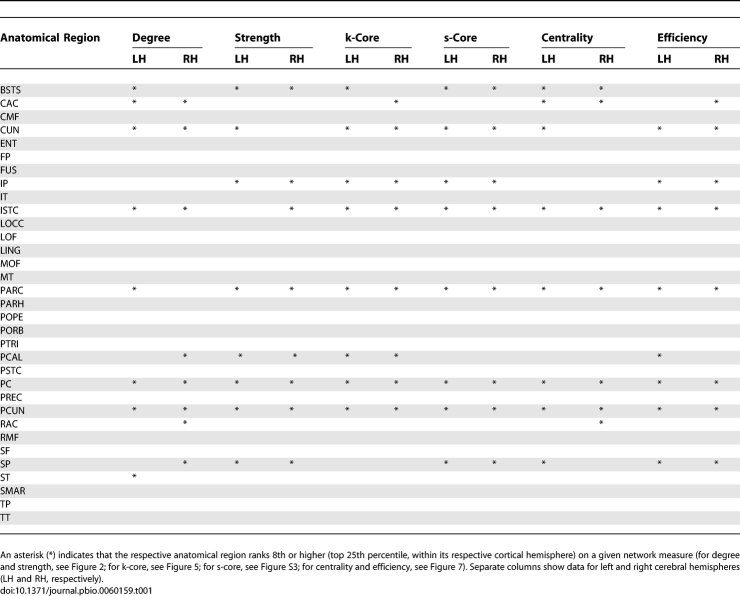
Summary of Data on Network Measures

The central structural embedding of posterior medial cortex in the human brain is consistent with a series of physiological findings including high levels of energy consumption and activation at rest [14] and significant deactivation during goal-directed tasks [13,14,17]. We found a significant positive correlation (*r* ^2^ = 0.49, *p* < 0.01, Figure S5) between centrality as reported in this paper and regional cerebral blood flow (rCBF) data from an earlier imaging study [14]. Studies of resting state functional networks have reported a high density of strong functional connections in posterior cortex [8]. In such networks, the precuneus was found to exhibit short path length, low clustering, and high centrality [8,11]. Activation of the precuneus [38] and of other cortical midline structures [39] has been linked to self-referential processing and consciousness. Reduced metabolic activation in the posterior cingulate cortex [40], amyloid deposition, and atrophy [41], as well as impaired task-dependent deactivation in posterior medial cortex, is associated with the onset of Alzheimer-type dementia [42,43].

The human default network comprises a set of interacting subsystems linked by hubs [44]. Key components of the default network are the posterior cingulate cortex, the precuneus, the lateral and medial parietal cortex, and the medial prefrontal cortex [12,13,15,17]. Of these areas, medial prefrontal cortex is the only component entirely excluded from the structural core. Our structural results suggest the hypothesis that default network activity may be driven from highly coupled areas of the posterior medial and parietal cortex, which in turn link to other highly connected and central regions, such as the medial orbitofrontal cortex. Consistent with this hypothesis, we found a close correspondence between the strengths of structural connections derived from DSI and functional connections derived from resting state fMRI in the same participants. Additional studies are needed to fully address the relationship between structural and functional connection patterns (Honey CJ, Sporns O, Cammoun L, Gigandet X, Meuli R, Hagmann P; unpublished data).

An important issue relates to the comparison of our present network analysis in human cortex to previous analyses carried out on anatomical connection matrices derived from tract-tracing studies in the macaque monkey. Direct comparison is made difficult by differences in spatial resolution (998 ROIs in human, 30–70 regions in macaque), the incomplete coverage of macaque cortex in most extant datasets, the lack of interhemispheric connections in the macaque, the lack of connection density data in the macaque, and the uncertainty of cross-species homologies between functionally defined brain regions [45]. A previous study focusing on the distribution of highly central hubs in macaque cortex had revealed the existence of connector hubs in some areas of prefrontal and parietal cortex [46], but was lacking connectional data on significant portions of posterior medial and frontal cortex (Figure S9). Here, we report ROIs with high centrality in several human cortical subregions, including medial and superior frontal cortex, inferior and superior parietal cortex, as well as cingulate and posterior medial cortex. The structural embedding of core regions within the human brain is consistent with anatomical studies of the connections of the macaque posteromedial cortex, which includes posterior cingulate and medial parietal regions. These regions are reported to have high interconnectivity as well as widespread connection patterns with other parts of the brain [47].

Previous attempts to provide a map of structural connections of the human brain have utilized correlations in cortical gray-matter thickness [48], as well as diffusion tensor imaging (DTI) [28,29]. Our approach to mapping human cortical structural connections was DSI followed by computational tractography [26,27]. DSI has been shown to be especially sensitive with regard to detecting fiber crossings. In macaque monkey [24], this method has been shown to produce connection patterns that substantially agree with traditional anatomical tract tracing studies. By extending these results, we found significant overlap between macaque connectivity data derived from DSI and from tract tracing (Text S4 and Figure S9). A more detailed mapping of the structural core in macaque will require the analysis of high-resolution DSI data from macaque cortex (Hagmann P, Gigandet X, Meuli R, Kötter R, Sporns O, Wedeen V; unpublished data). In human visual cortex, DSI connection patterns are in significant agreement with anatomical reports [27]. Furthermore, the high correlation of structural and functional connections patterns reported in this study, which holds for brain regions that are members of the structural core (e.g., the precuneus and posterior cingulate cortex, Figure 8B) as well as across the entire brain (Figure 8C), supports the validity of the DSI connectivity pattern. While these comparisons suggest that diffusion imaging can yield accurate connection maps, it must be noted that the method may be participant to scanning noise, errors in fiber reconstruction, and systematic detection biases. In particular, smaller fiber tracts and interhemispheric connections toward lateral cortices may be underrepresented given the limited resolution and complexity of the anatomy in the centrum semiovale. We note that our study focuses on a large-scale anatomical feature, the structural core, and that our main conclusions are insensitive to various degradations and manipulations of the original fiber density matrix (Text S3, Figure S6–S8).

Future improvements in diffusion imaging and tractography, as well as computational network analysis, will no doubt reveal additional features of the connectional anatomy of the human brain. It will be important to include major subcortical regions, such as the thalamus, into future network analyses. Another advance would be to parcellate cortex not on the basis of sulcal and gyral landmarks, but rather on the basis of regularities in functional connections that are observed in individual participants [49,50].

Our data provide evidence for the existence of a structural core in human cerebral cortex. This complex of densely connected regions in posterior medial cortex is both spatially and topologically central within the brain. Its anatomical correspondence with regions of high metabolic activity and with some elements of the human default network suggests that the core may be an important structural basis for shaping large-scale brain dynamics. The availability of single-participant structural and functional connection maps now provides the opportunity to investigate interparticipant connectional variability and to relate it to differences in individual functional connectivity and behavior.

## Methods

### 

#### Diffusion imaging and tractography.

The path from diffusion MRI to a high-resolution structural connection matrix of the entire brain consists of a five-step process (Figure 1): (1) diffusion spectrum and high resolution T1-weighted MRI acquisition of the brain, (2) segmentation of white and gray matter, (3) white matter tractography, (4) segmentation of the cortex into anatomical regions and subdivision into small ROIs, and (5) network construction.

#### Step 1: MRI acquisition.

After obtaining informed consent in accordance with our institutional guidelines, we scanned five healthy right-handed male volunteers aged between 24 and 32 y (mean = 29.4, S.D. = 3.4). Imaging was performed on an Achieva 3T Philips scanner using a diffusion weighted single-shot EPI sequence with a TR of 4,200 ms and a TE of 89 ms. The maximum diffusion gradient intensity was 80 mT/m, the gradient duration δ was 32.5 ms and the diffusion time Δ was 43.5, yielding a maximal b-value of 9,000 s/mm^2^. Q-space was sampled over 129 points located inside a hemispherical area of a cubic lattice, by varying the diffusion gradient intensity and direction such that **q** = *a* **q**_x_ + *b* **q**_y_ + *c* **q**_z_, (where *a*, *b*, and *c* are integers such that 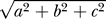 ≤ 4; **q**_x_, **q**_y_, and **q_z_** denote the unit diffusion sensitizing gradient vectors in the three respective coordinate directions; and **q** = γδ**g**_,_ where γ is the gyromagnetic ratio and **g** is the gradient strength (mT/m). The axial field of view was set to 224 by 224 mm and the acquisition matrix was 112 by 112, yielding an in-plane resolution of 2 × 2 mm. Parallel imaging was used with our eight-channel head coil with a reduction factor of 3. 36 contiguous slices of 3-mm thickness were acquired in two blocks resulting in an acquisition time of 18 minutes. In addition, a high resolution T1-weighted gradient echo sequence was acquired in a matrix of 512 × 512 × 128 voxels of isotropic 1-mm resolution.

Data reconstruction was performed according to a DSI protocol [26,27,51]. In every brain position, the diffusion probability density function (PDF) was reconstructed by taking the discrete 3D Fourier transform of the signal modulus symmetric around the center of q-space. The signal was pre-multiplied by a Hanning window before Fourier transformation in order to ensure smooth attenuation of the signal at high |**q|** values. The 3D PDF was normalized by dividing by its integral at every voxel. The orientation distribution function (ODF) φ was derived directly from the PDF by taking a radial summation of the 3D PDF *p*(*r*):

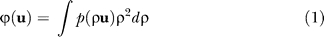
where ρ is the radius and **u** is a unit direction vector. The integral was evaluated as a discrete sum over the range ρ ∈ [0,5]. The ODF is defined on a discrete sphere and captures the diffusion “intensity” in every direction. It was evaluated for a set of vectors **u** *_i_* that are the vertices of a tessellated sphere with mean nearest-neighbor separation approximately 10°. The result was a diffusion map composed of ODFs at every location in the brain. The ODFs were represented as deformed spheres with the radius proportional to φ(**u**).

#### Step 2: White and gray matter segmentation.

The goal of the second step was two-fold as we wanted to obtain high-quality white matter segmentation for use in the tractography (step 4) as well as a high-quality segmentation of the cortex for use in the creation of the ROIs (step 3). Based of the high resolution T1w image, this step was performed in Freesurfer (http://surfer.nmr.mgh.harvard.edu) [52,53]. The output was an image with labels corresponding to the white matter, the cortex, and the deep cerebral nuclei.

#### Step 3: Creation of normalized cortical regions of interest.

One of the critical steps of the whole procedure was to partition the participants' cortex into ROIs located in an identical topographic position for each participant despite interindividual anatomical variation. We used Freesurfer to register a labeled mesh from an average brain onto the brain of each individual participant, where each label corresponded to one of 66 anatomical cortical regions [54]. This output provided for every participant a standardized partition of the cortex into 66 regional areas. In a second step, each of these regional areas were subdivided on the Freesurfer average brain into a set of small and compact regions of about 1.5 cm^2^, resulting in 998 ROIs covering the entire cortex. This subdivision was then registered on the individual brain using the same transformation as for the 66 regional areas thus maintaining the topological constraints of mapping. Consequently, the resulting partitions of the cortex into 66 and 998 ROIs were in anatomically closely matched positions for all participants (Cammoun L, Gigandet X, Thiran JP, Do KQ, Maeder P, et al., unpublished data).

#### Step 4: White matter tractography.

Tractography is a post-processing method that uses the diffusion map to construct 3D curves of maximal diffusion coherence. These curves, called *fibers*, are estimates of the real white matter axonal bundle trajectories [24,27]. Since DSI, in contrast to DTI, provides several directions of diffusion maximum per voxel, we modified the usual path integration method (deterministic streamline algorithm, [21,25]) to account for fiber crossings and to create a set of such fibers for the whole brain [24,27]. The methodology is summarized below:

*Detection of the Directions of Maximum Diffusion.* At each voxel, we defined a set of directions of maximum diffusion as local maxima of φ(**u**) (i.e., vectors **U** *_i_* such that φ(**u** *_j_*) *<* φ (**U** *_i_*) for all **u** *_j_* adjacent to **U** *_i_* in the sampled tessellated sphere. This step is equivalent to computing the principal eigenvector field in DTI.

*Fiber Computation.* We initiated the same number of fibers for every direction of maximum diffusion in every voxel of the segmented white matter. For example, in a voxel with two directions, we initiated 30 fibers along each direction, for a total of 60 fibers. The starting points were chosen spatially at random within the voxel. From each initialization point, a fiber trajectory is computed in a way similar to forming a streamline in a vector field with the additional constraint that in some locations, multiple orientations may occur. This is handled in the following way. From each initialization point, we began growing a fiber in two opposite directions with a fixed step of 1 mm. Upon entering a new voxel, the fiber growth continued along the direction of the vector **U** *_j_* (in the new voxel) whose orientation was the closest to the current direction of the fiber. If this resulted in a change of direction sharper than 30°/mm, the fiber was stopped. The growth process of a valid fiber finished when both its ends left the white matter mask. In this article we used about 3 million initialization points, of which between one-half and two-thirds connected cortical areas and were therefore retained.

#### Step 5: Network construction.

Finally, we combined the output of steps 3 and 4 and created the graph of brain structural connectivity. Every ROI constructed in step 3 became a node in the graph. We denoted by ROI(*v*) the ROI associated with node *v*. Its cortical surface was *S_v_*. Two nodes *v* and *u* were connected with an edge *e* = (*v*, *u*) if there was at least one fiber *f* with end-points in ROI(*v*) and ROI(*u*). For each edge *e*, we defined its length *l*(*e*) and weight *w*(*e*), as follows. Denoted by *F_e_* was the set of all fibers connecting ROI(*v*) and ROI(*u*) and hence contributing to the edge *e*. The length *l*(*e*) of the edge *e* was the average over the lengths of all fibers in *F_e_*, i.e., *l*(*e*) = 1/


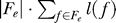
 , where *l*(*f*) is the length of fiber *f* along its trajectory. The weight *w*(*e*) captured the connection density (number of connections per unit surface) between the end-nodes of the edge *e*, and is defined as *w*(*e*) = 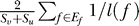 . The correction term *l*(*f*) in the denominator was needed to eliminate the linear bias towards longer fibers introduced by the tractography algorithm. The sum *S_v_ + S_v_* corrects for the slightly variable size of cortical ROIs.

The end result of this procedure was a weighted network of 998 ROIs of surface area approximately 1.5 cm^2^, covering the entire cortex and grouped into 66 anatomical subregions (for a list of abbreviations, see Figure 1). The anatomical positions of the ROIs were in register across participants, allowing for averaging across individual networks.

#### Functional neuroimaging.

We conducted two independent resting state fMRI imaging runs for each of the five participants for which structural imaging datasets were also acquired. The scans were performed on a Siemens Trio 3T system using a standard gradient echo sequence. We used an axial plane with a field of view of 211 × 211 mm and a matrix of 64 × 64 voxels, yielding an in-plane resolution of 3.3 × 3.3 mm^2^. 35 slices of 3-mm thickness and 10% gap where acquired. In order to reach a sampling rate of 0.5 Hz, we used a TR of 2,000 ms and a TE of 30 ms. The PAT factor was 3 and participants were scanned for 20 min in the first session and 15 min in the second session. Participants were instructed to remain alert and keep their eyes closed.

The fMRI raw data were registered and resampled onto the b0 image of the diffusion scan using the rigid body registration tool of SPM5 (http://www.fil.ion.ucl.ac.uk/spm/). Time series were computed for each of the 998 ROIs previously defined in step 3 of the diffusion imaging methods (see above). This was achieved by dilating each ROI with an isotropic structuring element of 19 voxels and computing the average signal intensity in the dilated ROI for every time point. The resulting time series were detrended, and the global brain signal was regressed out before computing cross-correlation maps. High-resolution (998 ROI) correlation maps were downsampled by averaging to yield correlation maps for 66 anatomical subregions.

#### Network analysis.

*Connectivity Backbone.* To visualize network layout and clusters, we derived the network's connectivity backbone [30]. First, a maximum spanning tree, which connects all nodes of the network such that the sum of its weights is maximal, was extracted. Additional edges were added in order of their weight until the average node degree was 4. The resulting network constituted the connectivity backbone of the connection matrix and was used for the network visualizations in Figure 3 and 4.

*k-Core Decomposition.* This decomposition method [33] involves the recursive pruning of those nodes with degree less than k. Applied to large networks the method yields cores of vertices that are mutually linked by at least k connections. We derived all k-cores for high-resolution connection matrices, whose top 10,000 fiber densities were converted to ones. We developed a related method, which we call s-core decomposition, that recursively prunes weakest nodes up to a strength s. The remaining core contains only nodes with strengths of at least s. For a series of discrete degrees k_i_ or strengths s_i_ we can then derive the corresponding k_i_-th and s_i_-th cores. Any of the 66 anatomical subregions was considered part of the k_i_-th or s_i_-th core if at least half of its ROIs were present in that core.

*Modularity Detection.* We applied a variant of a spectral community detection algorithm [34] to identify modules (communities) within each network. As inputs to the algorithm we used symmetric connectivity matrices corresponding to individual (998 ROI) or aggregated (66 anatomical region) fiber densities. The algorithm generated a modularity matrix with an associated modularity score. For regional connection matrices (*n* = 66), we obtained 10,000 solutions which were ranked according to their modularity and we selected the optimal solution for a range of 2–12 modules. For high-resolution matrices we obtained 20,000 solutions for modularity ranging from 3–8 modules.

*Hub Classification.* Cluster assignment from the optimal modularity matrices provided the basis for the classification of network hubs into two groups [35]. We calculated each node's participation index *P*, which expresses its distribution of intra- versus extra-modular connections. *P* of node *i* is defined as

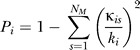
where *N_M_* is the number of identified modules, *k_i_* is the degree of node *i*, and κ_is_ is the number of edges from the *i*th node to nodes within module *s*. We classified nodes with above average degree and a participation coefficient *P* < 0.3 as provincial hubs, and nodes with *p* ≥ 0.3 as connector hubs.

*Graph Theory Methods.* With the sole exception of k-core decomposition and node degree, all graph theoretical analyses in this study were carried out for weighted networks. Node degrees were calculated as the column sums of the binarized connection matrix (i.e., the number of all edges for each node, regardless of weight). Node strengths were calculated as the column sum of the non-binarized connection matrix (i.e., the sum of all edge weights for each node).

Centrality of a node expresses its structural or functional importance. Highly central nodes may serve as waystations for network traffic or as centers of information integration. The betweenness centrality of a node is defined as the fraction of shortest paths between any pair of vertices that travel through the node [36]. The betweenness centrality of a node *i* is given as

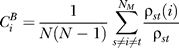
where ρ*_st_*(*i*) is the total number of shortest paths between a source node *s* and a target node *t* that pass through *i*, and ρ*_st_* is the total number of all shortest paths linking *s* to *t*.

Efficiency of a node is defined as the arithmetic mean of the inverses of the path lengths between the node and all other nodes in the network [11,55], i.e.,

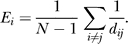



## Supporting Information

Figure S1 Degree and Strength Distributions (A) Degree distribution, averaged across all five participants.
(B) Linear-log plot of the cumulative node degree and node strength distributions, aggregated across all five participants. Red line indicates best linear fit, consistent with an exponential decrease of degree and strength towards higher values.
(409 KB TIF)

Figure S2 Individual Participant Variation Regional connection matrices for all five individual participants, plotted on a logarithmic scale. Note the high degree of correspondence between scans 1 and 2 of participant A, and between all other participants. Repeat scans for participant A are correlated with *r* ^2^ = 0.78, while the average between-participant correlation is *r* ^2^ = 0.65.
(4.06 MB TIF)

Figure S3 s-Core Decomposition s-Core decomposition was carried out as described in the Methods section of the paper. Individual s-core plots are shown for all five participants (A), as well as a plot showing the average s-core across all five participants (B). (C) A rank-ordered distribution of s-core numbers for all 66 anatomical subregions is shown.
(1.34 MB TIF)

Figure S4 Cluster Analysis of Visual and Frontal Cortex (A) After selection of visual ROIs and their interconnections, we searched for optimal modularity using the spectral community detection algorithm described in [34]. The plot shows an overlay of the cluster arrangement found for each of the five participants. Red, green, and blue dots indicate the anatomical positions of ROIs grouped into three distinct clusters, roughly corresponding to occipital, ventral, and dorsal visual system, respectively. Pure colors indicate that the ROI was grouped consistently (for all five participants) into the corresponding cluster; intermediate clusters indicate groupings that were inconsistent across participants.
(B) Clusters obtained after modularity analysis restricted to frontal ROIs and their interconnections, roughly corresponding to medial, lateral, and orbital frontal cortex.
(912 KB TIF)

Figure S5 Centrality and Regional Cerebral Blood Flow Regional cerebral blood flow data were obtained from Table 1 of [14]. Regional designations refer to medial, right, or left cortices, and Brodmann areas. Centrality data was computed as the average centrality of the five ROIs located closest to the Talairach coordinate provided in Table 1 of [14]. All ROIs were within 12 mm of the target coordinate. The correlation between rCBF and centrality is *r* ^2^ = 0.49, *p* < 0.01. ROIs for the three regions with highest rCBF (M31/7, M10, M32) were located in these anatomical subregions: right precuneus, right and left rostral anterior cingulate cortex, and right and left medial orbitofrontal cortex. Very similar correlations were found between centrality and data for the cerebral metabolic rate for oxygen [14], as well as data from a second participant group (Table 2 of [14]).
(557 KB TIF)

Figure S6 Robustness of Centrality Estimates Each panel shows a rank-ordered distribution of centrality for anatomical subregions in left and right hemisphere, after the high-resolution connection matrix was subject to a random perturbation. All distributions are for *n* = 10 separate perturbations for each participant, followed by averaging over all five participants. (A) Ten percent of all edges were randomly rewired. (B) Ten percent of edge weight was either added or subtracted from all edges. (C) The edges for 100 randomly selected pairs of ROIs were swapped.
(1.25 MB TIF)

Figure S7 Node Strength and Centrality for Regional Connection Matrices Node strength (A) and centrality (B) for regional connection matrices obtained from each of the five participants. Plots show rank-ordered distributions.
(721 KB TIF)

Figure S8 Network Measures after Edge Weight Transformation Node strength (A) and centrality (B) for high-resolution connection matrices whose edge weights were transformed to a Gaussian distribution (see Text S3 for details). (C) Community structure (optimal modularity and hubs) for an average regional connection matrix obtained after edge weights were resampled to a Gaussian distribution. Plotting conventions are as in Figure 6.
(4.7 MB TIF)

Figure S9 Comparison of Macaque Cortex Structural Connections Derived by Diffusion Imaging and Tractography (A) Composite matrix of DSI-derived fiber densities (lower triangular) and symmetrized anatomical connection matrix (upper triangular) derived from Cocomac data (http://www.cocomac.org/). Fiber densities in the lower triangular portion of the matrix are displayed on a proportional gray scale (arbitrary units), while Cocomac pathways in the upper triangular matrix are displayed as “known present” (black), “unknown” (gray), and “known absent” (white). Two main clusters of brain regions, derived by cluster analysis of functional connectivity (see [20]) are color-coded in blue (mostly containing occipitotemporal areas) and green (mostly containing parietofrontal areas). Their anatomical locations, as well as the extent to which the matrix covers the surface of macaque cortex are shown in the panels at the top of the figure (lateral and medial views, respectively). (B) Proportions of the total DSI fiber mass that are coinciding with “known present,” “unknown,” and “known absent” Cocomac pathways.
(1.83 MB TIF)

Table S1 Modularity (28 KB DOC)

Table S2 Talairach Coordinates of ROIs with High Centrality (66 KB DOC)

Text S1 Assortativity (24 KB DOC)

Text S2 Small-World Attributes and Structural Motifs (66 KB DOC)

Text S3 Robustness of Graph Measures (27 KB DOC)

Text S4 Macaque Diffusion Imaging (36 KB DOC)

## Abbreviations

DSI: diffusion spectrum imaging
DTI: diffusion tensor imaging
MRI: magnetic resonance imaging
PDF: probability density function
rCBF: regional cerebral blood flow
ROI: region of interest
ODF: orientation distribution function
