# Supplementary material for: Mapping the Structural Core of Human Cerebral Cortex
Source: PLoS Biol. 2008 Jul 1;6(7):e159. doi: 10.1371/journal.pbio.0060159 (PMC2443193; doi:10.1371/journal.pbio.0060159)
Supplement: Supplementary file 1 [file pbio.0060159.sd001.doc]

**Text S1: Assortativity**

ROIs with high degree or strength may connect randomly or they may connect preferentially to one another. We examined high-resolution connection patterns from individual subjects for degree and strength correlations by computing the network’s assortativity [1]. We found that networks of all five subjects exhibited positive assortativity (0.1941 +/- 0.0513 for degrees; 0.2161 +/- 0.0275 for strengths) indicating a tendency for nodes with matching degree or strength to be directly connected. As expected, degree-matched randomized control networks showed assortativity narrowly distributed around zero (e.g. -0.0122 +/- 0.0098 for degrees). Positive assortativity is consistent with the existence of a densely interconnected and coherent structural core of brain regions.

Reference:

1. Newman, MEJ (2002) Assortative mixing in networks. Phys. Rev. Lett. 89, 208701.
