# Supplementary material for: Mapping the Structural Core of Human Cerebral Cortex
Source: PLoS Biol. 2008 Jul 1;6(7):e159. doi: 10.1371/journal.pbio.0060159 (PMC2443193; doi:10.1371/journal.pbio.0060159)
Supplement: Supplementary file 2 [file pbio.0060159.sd002.doc]

**Text S2: Small-World Attributes and Structural Motifs**

Clustering coefficients were calculated using the method by Onnela et al. [1] which defines clustering in weighted networks as the sum of all triangle intensities surrounding a central node i:

$$C_{i} =\frac{2}{k_{i}(k_{i}-1)}\sum_{j,k} (w_{ij}w_{jk}w_{ki})^{1/3},$$

where *k_i_* is the degree of the *i*-th node and *w*_ij_ is the weight of the connection from *i* to *j* normalized by dividing by the largest edge-weight in the network.

Path lengths are calculated using Dijkstra’s algorithm on the weighted network after transforming the connection weights (fiber densities) to distances using the formula $d_{ij} =w_{ij}^{-1}-1.$

The small-world index was derived by a comparison of scaled clustering coefficients and path lengths, as described by Humphries et al. [2]. Network topology may be said to correspond to a “small world” [3] if the network’s clustering coefficient is much greater than that of equivalent random controls $\gamma>>\gamma_{random}$, while their path lengths are comparable $\lambda\approx\lambda_{random}$. The small-world index $\sigma_{sw}$is defined as:

$$\sigma_{sw} =\frac{\gamma/\gamma_{random}}{\lambda/\lambda_{random}}$$

Comparisons are carried out against populations of n = 100 degree-matched randomized control networks.

Motifs were counted using the approach by Onnela et al. [1]. For the present study we only counted triangles, i.e. fully connected motifs of size 3, and computed z-scores for motif intensity and coherence by comparing experimental networks to populations of 100 randomized degree-matched controls. These control networks preserved degree sequence and weight distributions (but not weight sequences) of the experimental networks. Additional comparisons were carried out in some cases for randomized networks that preserved both node degree and (approximately) node strength. Comparisons across both random populations led to very similar results.

We computed clustering coefficients and path lengths for each individual high-resolution connection matrix, and performed comparisons of these measures to populations (n = 100) of randomized degree-matched controls. For the five individual subjects, the average small-world index was 10.64 (+/- 0.61), indicative of a high degree of small-world organization, as previously described for thresholded networks [4]. As expected, the set of regional connection matrices for the five subjects exhibited much smaller values of the small-world index, with a mean of 1.54 (+/- 0.23). These values are within the same range as those previously reported for similar-sized binary anatomical matrices from macaque and cat cortex [5].

Our results for clustering coefficients are consistent with the abundance of specific weighted motifs. Average motif intensities and coherences for a fully connected 3-node motif are significantly increased over those found in randomized controls, for all five subjects (motif z-scores > 500 for intensity and coherence, all comparisons relative to degree–matched randomized controls, data not shown).

References:

1. Onnela, JP, Saramaki, J., Kertesz, J., Kaski, K. (2005) Intensity and coherence of motifs in weighted complex networks. Phys. Rev. E 71, 065103.

2. Humphries, MD, Gurney, K, Prescott, TJ (2006) The brainstem reticular formation is a small-world, not scale-free, network. Proc Roy Soc B Biol Sci 273: 503-511.

3. Watts, DJ, Strogatz, SH (1998) Collective dynamics of ‘small-world’ networks. Nature 393: 440-442.

4. Hagmann P, Kurant M, Gigandet X, Thiran P, Wedeen VJ, et al. (2007) Mapping human whole-brain structural networks with diffusion MRI. PLoS ONE 2: e597.

5. Sporns, O, Zwi, J (2004) The small world of the cerebral cortex. Neuroinformatics 2, 145-162
