# Supplementary material for: Mapping the Structural Core of Human Cerebral Cortex
Source: PLoS Biol. 2008 Jul 1;6(7):e159. doi: 10.1371/journal.pbio.0060159 (PMC2443193; doi:10.1371/journal.pbio.0060159)
Supplement: Supplementary file 3 [file pbio.0060159.sd003.doc]

**Text S3: Robustness of Graph Measures**

A systematic assessment of inter-subject and inter-hemispheric differences requires larger sample sizes and was therefore not attempted in the current study. Nonetheless, we note that regional connection matrices for all five subjects were highly correlated (Spearman’s rank correlation, mean r^2^ for inter-subject comparisons = 0.65; cf. Figure S2), with two independent scans of subject A showing even greater consistency (r^2^ = 0.78). This indicates that regional connection matrices can be obtained from single subjects with high accuracy and repeatability across scans. As expected, high reproducibility of edge weights translates into reproducible network measures. For example, centrality (Figure 7C) was consistent between the two scans for subject A (r^2^ = 0.54) as well as between subjects (r^2^ = 0.42). When considering centrality for the 12 largest anatomical subregions (consisting of the largest number of ROIs and thus least affected by variability in ROI alignment) the within-subject correlation increased to r^2^ = 0.70.

To test the robustness of our results relative to random variations in the fiber density matrix, we performed three sets of control analyses. First, we rewired 10% of all edges by swapping the edge weights of randomly selected pairs. Second, we added or subtracted 10% of the weight of each edge. Third, we swapped all edges for 100 randomly selected pairs of nodes. Each of these manipulations resulted in a degraded or randomized connection matrix, for which all network indices were obtained. For example, when examining centrality for each of these three manipulations and across all five subjects we find that the regional rank orderings (Figure S6) closely correspond to those obtained for the non-degraded networks (Figure 7C). For example, the posterior cingulate cortex and the precuneus remain at or near the top of the distribution for all three manipulations. This indicates that our main conclusions are unaffected by small variations in edge weights or topology possibly introduced by imaging noise or tractography errors.

Most network measures reported in this study have been obtained from the weighted high-resolution connection matrices (N=998) followed by averaging over anatomical subregions and subjects. Application of network measures directly to individual regional matrices (N=66) confirms our main results (Figure S7).

Fiber densities are not equal to physiological strengths or efficacies. Since diffusion imaging does not allow the direct measurement of the physiological efficacies of fiber pathways, we used a simple heuristic to map fiber densities (which range over several orders of magnitude) onto connection efficacies (which are likely to show a more narrow distribution). We resampled fiber densities to a Gaussian distribution with a mean of 0.5 and a standard deviation of 0.12, which results in normally distributed connection weights that retain their rank. This transformation assigns high physiological efficacy to dense fiber pathways, while drastically compressing the range of edge weights. We find little change in rank-ordered distributions of node strengths and centrality (Figure S8A,B). The community structure consists of 6 modules largely corresponding to those described earlier for the fiber density network (Figure S8C). The set of connector hubs is found predominantly along the medial axis and includes several of the anatomical subregions described above (Figure 6A) as well as medial orbitofrontal cortex and the frontal poles. We conclude that re-scaling of edge weights from raw fiber densities to a more physiological range has little impact on the conclusions of this study.
