# Supplementary material for: Mapping the Structural Core of Human Cerebral Cortex
Source: PLoS Biol. 2008 Jul 1;6(7):e159. doi: 10.1371/journal.pbio.0060159 (PMC2443193; doi:10.1371/journal.pbio.0060159)
Supplement: Supplementary file 4 [file pbio.0060159.sd004.doc]

**Text S4: Macaque Diffusion Imaging**

**Histologic Tracing.** The macaque neocortex is parcellated according to the original Felleman and Van Essen paper [1] and includes visual, somatosensory and motor cortical regions. The connections between these regions were manually collated in the CoCoMac database from published tracing [2]. Subsequently, all relevant data were translated algorithmically to the Felleman and Van Essen cortical map using coordinate-independent mapping. After resolution of redundant and inconsistent results a binary connection matrix with 47 nodes (vertices) and 505 edges (connections) was generated. For a more complete structural description, see a previous report [3]. To make it compatible with DSI connectivity data, which is undirected, the matrix was symmetrized (Figure S9A), resulting in a 47 by 47 connection matrix with 313 pathways that had been anatomically traced and reported as present (“known present”), while the remainder of the pathways were either unreported (“unknown”) or had been reported as absent (“known absent”).

**MR Tractography.** Imaging was performed on one hemisphere of a healthy adult male *M. fascicularis* monkey. After euthanasia, the brain was fixed with formalin for at least 4 weeks. Then the brain was soaked for approximately one month in a buffer solution containing 2 mM gadolinium (Gd-DTPA) and scanned on a 4.7 T Bruker Biospec system. The pulse sequence was a 3D diffusion weighted spin-echo EPI sequence, with TR/TE 450/63 ms, with an imaging matrix of 128 by 112 by 80 voxels with isotropic spatial resolution of 500 µm^3^. Diffusion spectrum encoding was performed as previously described in [4]. It consisted of 515 diffusion weighted measurements, corresponding to points in a cubic lattice of Q-space contained within the interior of a ball of maximum radius bmax = 4 • 10^4^ cm^2^/s. Diffusion encoding gradients lasted for δ = 12 ms and were separated by a diffusion time of Δ = 31 ms, and with peak amplitude 400 mT/m. Number of averages per gradient direction was 4. Total acquisition time was 25 h. Diffusion spectra were reconstructed in the same way as the human data reported in this study and following [4]. To obtain the 3D diffusion pdf a 3D Fourier transform of the modulus of the acquired data is computed in every voxel. Whole (hemi)-brain DSI streamline tractography was performed in an identical way as for the human data reported in this study [5]. The hemispheric cortex was partitioned into 1000 ROIs of equal size as described in [5]. A 1000 by 1000 connection matrix was then built by capturing the fibers ending in each pair of ROIs and the connection density was calculated. Then manually these small ROIs were grouped to match the Van Essen cortical partition as depicted in Figure S9A. The mean connection density between these cortical regions was calculated and reported in a 47 by 47 regional connection matrix (Figure S9A).

References

1. Felleman DJ, Van Essen DC (1991) Distributed hierarchical processing in the primate cerebral cortex. Cereb Cortex 1: 1-47.

2. Kötter R. Online retrieval, processing, and visualization of primate connectivity data from the CoCoMac database. Neuroinformatics. 2004;2(2):127-44.

3. Honey CJ, Kötter R, Breakspear M, Sporns O (2007) Network structure of cerebral cortex shapes functional connectivity on multiple time scales. Proc. Natl. Acad. Sci. USA 104: 10240-10245.

4. Wedeen VJ, Hagmann P, Tseng WY, Reese TG, Weisskoff RM (2005) Mapping complex tissue architecture with diffusion spectrum magnetic resonance imaging. Magn Reson Med 54, 1377-1386.

5. Hagmann P, Kurant M, Gigandet X, Thiran P, Wedeen VJ, et al. (2007) Mapping human whole-brain structural networks with diffusion MRI. PLoS ONE 2, e597.
