# Supplementary material for: Mapping the Structural Core of Human Cerebral Cortex
Source: PLoS Biol. 2008 Jul 1;6(7):e159. doi: 10.1371/journal.pbio.0060159 (PMC2443193; doi:10.1371/journal.pbio.0060159)
Supplement: Supplementary file 5 [file pbio.0060159.st001.doc]

**Table S1: Modularity.**

Modules and member regions corresponding to those shown in Fig. 6A of the main text.

| Module Number | Member Regions (LH) | Member Regions (RH) |
| --- | --- | --- |
| 1 | lCUN, , lLING, lPARH, lPCAL, lPCUN | rCUN, rLING, rPCAL |
| 2 | lCAC, lISTC, lPARC ,lPC | rCAC, rISTC, rPARC, rPC, rPCUN |
| 3 | lBSTS, lENT, lFUS, lIP, lIT, lLOCC, lMT, lPSTC, lSP, lST, lSMAR, lTP, lTT |  |
| 4 |  | rBSTS, rENT, rFUS, rIP, rIT, rLOCC ,rMT ,rPARH, rPSTC, rSP, rST, rSMAR, rTP, rTT |
| 5 | lCMF, lFP, lLOF, lMOF, lPOPE, lPORB, lPTRI, lPREC, lRAC, lRMF, lSF |  |
| 6 |  | rCMF, rFP, rLOF, rMOF, rPOPE, rPORB, rPTRI, rPREC, rRAC, rRMF, rSF |
