# Supplementary material for: Mapping the Structural Core of Human Cerebral Cortex
Source: PLoS Biol. 2008 Jul 1;6(7):e159. doi: 10.1371/journal.pbio.0060159 (PMC2443193; doi:10.1371/journal.pbio.0060159)
Supplement: Supplementary file 6 [file pbio.0060159.st002.doc]

**Table S2: Talairach Coordinates of ROIs with High Centrality.**

List of Talairach coordinates (x, y, z) of ROIs with high centrality. ROIs were selected by averaging ROI centrality across all five subjects and selecting the top 5% (50 ROIs) across both hemispheres.

Right cerebral hemisphere:

| x | y | z | anatomical region |
| --- | --- | --- | --- |
| 25 | 1 | 45 | rCMF |
| 36 | 8 | 35 | rCMF |
| 5 | -77 | 25 | rCUN |
| 36 | -82 | 24 | rIP |
| 47 | -51 | 22 | rIP |
| 33 | -78 | 36 | rIP |
| 6 | -46 | 30 | rISTC |
| 10 | -40 | 33 | rISTC |
| 6 | -37 | 42 | rISTC |
| 8 | 26 | -11 | rMOF |
| 6 | -20 | 50 | rPARC |
| 9 | -7 | 40 | rPC |
| 3 | -25 | 32 | rPC |
| 7 | -17 | 38 | rPC |
| 27 | -9 | 45 | rPREC |
| 11 | -47 | 64 | rPCUN |
| 6 | -58 | 16 | rPCUN |
| 15 | -44 | 49 | rPCUN |
| 7 | -52 | 45 | rPCUN |
| 7 | -42 | 44 | rPCUN |
| 7 | -62 | 48 | rPCUN |
| 3 | -65 | 31 | rPCUN |
| 4 | -66 | 39 | rPCUN |
| 8 | 37 | 14 | rRAC |
| 6 | 30 | -6 | rRAC |
| 11 | -1 | 45 | rSF |
| 5 | -6 | 51 | rSF |
| 12 | 13 | 39 | rSF |
| 19 | -83 | 37 | rSP |
| 16 | -74 | 45 | rSP |
| 15 | -64 | 48 | rSP |

Left cerebral hemisphere:

| x | y | z | anatomical region |
| --- | --- | --- | --- |
| -7 | 18 | 30 | lCAC |
| -6 | -88 | 12 | lCUN |
| -6 | -75 | 22 | lCUN |
| -12 | -88 | 27 | lCUN |
| -6 | -45 | 30 | lISTC |
| -6 | -15 | 52 | lPARC |
| -18 | -39 | 50 | lPARC |
| -18 | -26 | 39 | lPARC |
| -7 | -74 | 16 | lPCAL |
| -31 | -34 | 51 | lPSTC |
| -42 | -27 | 45 | lPSTC |
| -12 | -9 | 39 | lPC |
| -14 | -19 | 37 | lPC |
| -6 | -24 | 38 | lPC |
| -8 | -41 | 41 | lPCUN |
| -46 | 29 | 29 | lRMF |
| -10 | -5 | 48 | lSF |
| -36 | -43 | 49 | lSP |
| -24 | -58 | 58 | lSP |
